# Challenges and Lessons Learned From a Telehealth Community Paramedicine Program for the Prevention of Hypoglycemia: Pre-Post Pilot Feasibility Study

**DOI:** 10.2196/26941

**Published:** 2021-08-03

**Authors:** Mohanraj Thirumalai, Ayse G Zengul, Eric Evans

**Affiliations:** 1 Department of Health Services Administration University of Alabama at Birmingham Birmingham, AL United States

**Keywords:** hypoglycemia, telehealth, community paramedicine, diabetes, self-efficacy

## Abstract

**Background:**

Prevention through Intervention is a community paramedicine program developed by Birmingham Fire and Rescue Services in Alabama. This program aims to reduce dependency on emergency medical services (EMS) for nonemergency-related events through education and to lower the frequency of emergency calls in underserved populations. A telehealth intervention with an emphasis on hypoglycemia was implemented to (1) tailor the intervention to meet the educational needs of participants and (2) facilitate follow-ups. A pre-post pilot feasibility evaluation of the telehealth intervention was conducted.

**Objective:**

This paper describes the results of the feasibility evaluation, implementation challenges, and the lessons learned about the deployment of a hypoglycemia prevention program in an underserved area and its evaluation.

**Methods:**

This single-arm pretest-posttest intervention included (1) an initial in-person visit (week 1), (2) 3 weekly telecoaching calls (weeks 2-4), (3) 1 biweekly call (week 6), and (4) a final in-person visit (week 8) for collecting posttest data from individuals who called EMS due to hypoglycemic events. In-person visits included educational sessions conducted by EMS personnel. Participants’ education included tailored content related to hypoglycemia. Weekly telecoaching calls focused on hypoglycemia symptom monitoring and education reinforcement via a telehealth dashboard. The primary measures focused on feasibility measures, and exploratory measures focused on the fear of hypoglycemia, self-efficacy, and a knowledge of diabetes.

**Results:**

A total of 40 participants participated in the intervention. However, the study was marred with high attrition. The various factors behind the low retention rate were discussed. There was a decreasing trend in all three subdomains of the fear of hypoglycemia from pretest to posttest. There was also a significant increase in participants’ self-efficacy in hypoglycemia self-management (*P*=.03).

**Conclusions:**

This study shows preliminary and promising results for a community-based intervention specifically for hypoglycemia. However, the socioeconomic setting in which the intervention was delivered may have resulted in high dropout rates and low attendance during the intervention, which are considerations for future telehealth studies.

**Trial Registration:**

ClinicalTrials.gov NCT03665870; https://clinicaltrials.gov/ct2/show/NCT03665870

## Introduction

Hypoglycemia is a common but potentially avoidable health problem that can be a barrier to achieving good glycemic control. Hypoglycemia is indicated by abnormally low blood glucose concentrations (usually <70 mg/dl) and can result from physical exercise, certain diets, the misuse of drugs, endocrine disorders, and renal insufficiency [1,2]. However, antidiabetic agents, which increase insulin production and exogenous insulin levels, are the most common causes of hypoglycemia [3,4]. Although mild episodes of hypoglycemia occur 0.8 to 2 times per week in people with type 1 diabetes and people with insulin-treated type 2 diabetes, severe hypoglycemia rates range from 1.4 to 1.7 episodes per year. Except for some severe events, the majority of hypoglycemic episodes can be prevented and easily treated at home by following simple guidelines [2,5]. If it is not treated, hypoglycemia can have deleterious effects on people’s quality of life [6], mortality, and morbidity [7,8]. The unpleasant aspects of hypoglycemia may result in severe anxiety and the fear of hypoglycemia (FH) in people with diabetes. The FH is associated with the frequency of past hypoglycemic episodes and can promote compensatory behaviors, such as reducing insulin dosages to avoid hypoglycemia. This can be a major barrier to achieving glycemic control for people with diabetes [9-11]. In the prevention of hypoglycemia, sociocultural health literacy and the economic status of the population are critical components that necessitate the education of health care practitioners and patients—a key factor in the provision of care [12].

Inadequate health literacy is common among vulnerable populations. It is independently associated with poor glycemic control and an increased incidence of hypoglycemia (59%) in patients with diabetes [13,14]. It is also linked to the higher use of health care services, which costs the US economy between US $106 billion and US $238 billion annually [15]. The residents of the area served by our community paramedicine program belong to the lowest quartile of health literacy scores [16]. The initiation of the community paramedicine program offers an opportunity to improve care management and health literacy for patients with hypoglycemia. However, although studies on community education programs have reported successful outcomes [17-19], these studies mainly depend on the skill and knowledge of select individuals and have not resulted in the sustained integration of hypoglycemia interventions into regular practice.

As a model of mobile integrated health care programs, community paramedicine is an evolving community-based health care design that ultimately aims to increase access to basic paramedic services by integrating the services of multiple disciplines [20-22]. Through partnerships with local emergency medical services (EMS) and other health care services, mobile integrated health care–based community paramedicine programs deploy trained paramedics to help patients with complex chronic conditions at home. By visiting frequent users of the 911 system, these programs reduce the number of unnecessary emergency department transports and the number of nonemergency phone calls, thereby improving care management through patient education, advocacy, and navigation [23,24]. Fire departments receive thousands of “essentially preventable” medical emergency calls related to chronic conditions, including hypoglycemia [25-27].

A community paramedicine program, Prevention through Intervention, was initiated by the Birmingham Fire and Rescue Department in Alabama. This community paramedicine program attempts to expand access to health services for underserved rural populations who lack consistent primary care or preventive services and therefore frequently seek nonurgent care. The program involves educational home visits that are conducted by 1 paramedic who is assigned full-time to the program and provides services such as wellness and medication checks, safety assessments, and services for connecting people to primary care when such care is needed. This study was conducted as part of the Prevention through Intervention program. This study aimed to use telehealth to facilitate the tailoring of a telehealth intervention to meet the precise educational needs of participants, enable follow-ups, and perform a preliminary pilot feasibility and acceptability evaluation of the program.

## Methods

### Study Design

A single-arm pretest**-**posttest intervention group was used to test various outcomes (see *Measures* section). Due to our community partner expressing ethical concerns about including a control group without an intervention, it was not possible to establish an untreated control group. Therefore, a pretest**-**posttest design was chosen. This was also recommended by our community partner—the Fire and Rescue Department (ie, where this study was conducted). A total of 40 people enrolled in this study.

### Participant Eligibility, Recruitment, and Enrollment

The home of the community paramedicine program, which was where this study was conducted, receives about 1000 hypoglycemia-related EMS calls on an annual basis. All patients who called 911 due to hypoglycemia-related events were screened based on the following inclusion criteria: (1) residents in the service area of the fire district, (2) individuals aged ≥18 years, (3) individuals receiving intravenous 50% dextrose (intravenous treatment for hypoglycemia provided by EMS personnel), and (4) individuals who are not enrolled in any diabetes-related educational programs. All successfully screened participants were provided with an informed consent form. Participants were only enrolled in this study after they provided consent. Any resident who met the first 3 inclusion criteria based on EMS records (prescreening) was contacted via telephone by EMS personnel to assess their interest in participating in this study. If they were interested and were successfully screened for the last inclusion criterion, the resident was scheduled for an in-person consenting and baseline data collection session. This was done sequentially until 40 participants were enrolled in this study.

Unlike paramedics, emergency medical technicians in fire departments are not allowed to administer glucagon in prehospital settings [28]. They usually respond to hypoglycemic events by using intravenous 50% dextrose. Therefore, we did not include glucagon administration as part of the inclusion criteria.

### Intervention

**Figure 1:**
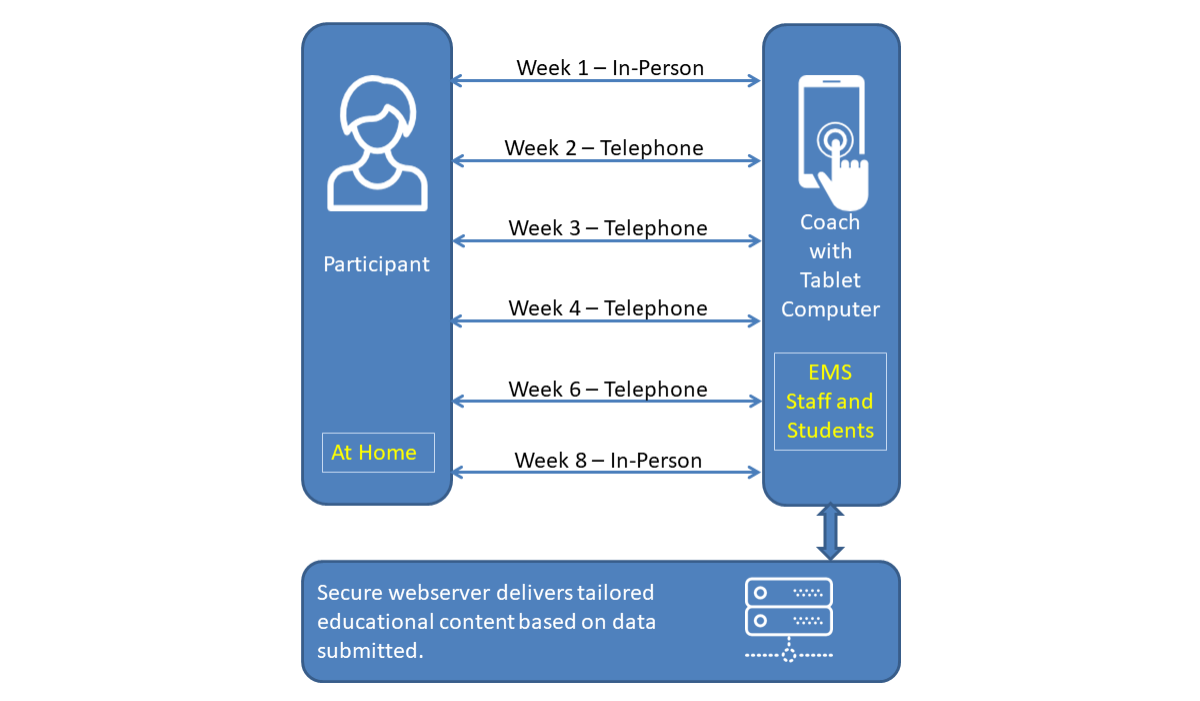
Study intervention design. EMS: emergency medical services.

The intervention included an initial in-person visit (week 1), which was followed by 3 weekly telecoaching calls (weeks 2-4), 1 biweekly call (week 6), and a final in-person visit in week 8 to perform posttest data collection (Figure 1). Anecdotal evidence from our community partner and related literature [29] indicated that having a lack of blood sugar and other vital sign monitoring devices and not visiting primary care physicians (to reevaluate prescriptions) often contributed to hypoglycemia episodes. To encounter these scenarios, health and wellness kits that included blood glucose meters, a blood pressure monitor, and oral dextrose gels were provided as an incentive to the participants.

During weeks 1 and 6 of this study, EMS personnel visited the homes of the recruited participants. During the week 1 visit, the EMS personnel used tablet computers to educate the participants. Their education involved the retrieval of tailored multimedia content, which was shown as a part of the verbal education provided on topics related to hypoglycemia. Based on an assessment of participants’ diabetes literacy, numeracy [30], and knowledge [31], appropriately tailored content that matched participants’ needs from the Diabetes Literacy and Numeracy Education Toolkit (DLNET) was provided by the telehealth platform. The DLNET is a comprehensive platform that was designed to facilitate diabetes education for patients with diabetes, especially for those with low health literacy. The DLNET provides 24 interactive modules that consist of diabetes care topics, such as blood glucose monitoring, exercise, and dietary instructions.

Student volunteers and EMS personnel used a telehealth dashboard, which was designed for this study, to coach and monitor participants over the phone during weeks 2, 3, 4, and 6. These calls focused on the active monitoring of hypoglycemia-related symptoms and the reinforcement of any education that participants received during week 1.

### Telehealth Platform

**Figure 2:**
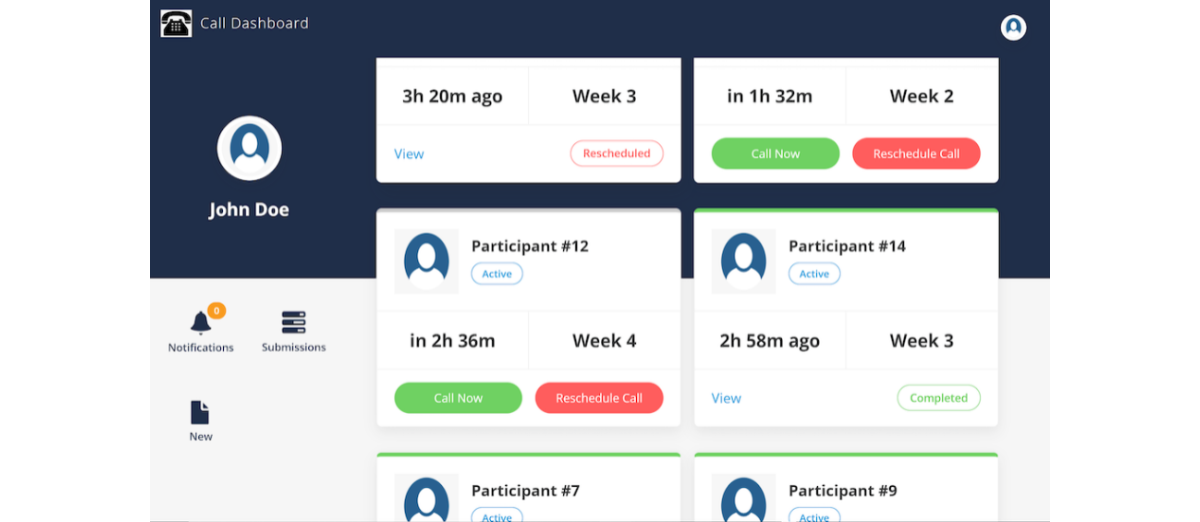
Telehealth dashboard.

The telehealth platform was built by repurposing, refining, and customizing a proven technical infrastructure that is currently being used by multiple projects (Figure 2). The platform used an Apache server (Apache Software Foundation) that runs a CakePHP back end that is connected to an Angular/Ionic framework–based front end. This enabled the telehealth platform to be delivered as both a web application and a hybrid mobile app with very minimal changes.

The telehealth dashboard automatically scheduled all of the recurring coaching calls for times that were convenient to the participants and in line with the intervention protocol. The community paramedicine personnel received alerts when it was time to call a participant and were able to mark the success or failure of completing the calls. When the calls were not successful, the calls could be rescheduled. It was also possible to perform weekly data collection and take notes during calls.

### Measures

#### Primary Measures

The primary focus of this study was evaluating the feasibility of recruitment, intervention delivery, retention, and data collection.

#### Exploratory Outcome Measures

One of the most important impacts of hypoglycemia is noncompliance with diabetes treatment due to the FH. This was measured by using the Hypoglycemia Scale: FH-15 questionnaire, which includes 15 items (5-point Likert scale) [32]. The response options were 1 (never), 2 (almost never), 3 (sometimes), 4 (almost always), and 5 (every day). A total score of ≥28 indicated that participants had an FH.

Self-efficacy in hypoglycemia self-management was assessed with the Perceived Diabetes Self-Management Scale (PDSMS) [33]. The questionnaire includes 8 items that are rated on a Likert 5-point scale (1=‘‘Strongly Disagree’’; 5=‘‘Strongly Agree’’). Higher scores represent higher levels of self-efficacy of hypoglycemia.

The Spoken Knowledge in Low Literacy Diabetes (SKILLD) [31] scale was used to assess participants’ diabetes knowledge. This 10-item questionnaire includes diabetes self-care–related questions, such as questions about glucose management and lifestyle modifications, for evaluating diabetes knowledge.

### Data Collection

All data pertaining to the exploratory measures were collected during the in-person visits conducted in week 1 and week 8 of this study. The data were directly entered into the tablet computer that was carried by the EMS personnel.

### Statistical Analysis

Participant attrition, session attendance, and overall instrument completion were recorded and analyzed by using descriptive statistics. We tested the pre-post exploratory measures by using simple parametric tests (one-tailed Student *t* test) after testing the normality of the data obtained. All statistical analyses were conducted by using SAS 9.4 (SAS Institute), and statistical significance was set at *P*<.05.

## Results

### Summary of Results

The findings from this study fell into 2 categories. First, we focused on the feasibility-related aspects of this study. Second, we focused on the exploratory outcomes. The lessons learned and challenges in implementing this study are presented in the *Discussion* section.

A total of 40 participants enrolled in this study. The mean age of participants was 67.13 years. The average age of males (n=18) was 69.33 years, and the average age of females (n=22) was 65.32 years.

### Feasibility Metrics

#### Recruitment

**Figure 3:**
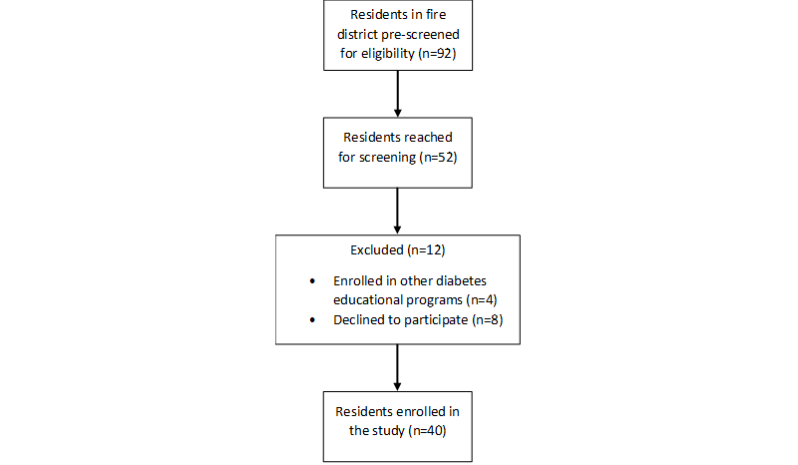
Participant enrollment flowchart.

For recruitment, we relied on EMS personnel to review EMS records and screen participants. Of the 92 people who were identified (prescreened) in a 6-month period, we were able to contact 52 (57%). Of the 52 people contacted, 40 (77%) fully qualified for and agreed to participate in this study (Figure 3).

#### Intervention Delivery and Retention

The first step of the intervention was an in-person visit, which was by the EMS personnel, to the participants’ homes. This session focused on obtaining consent, collecting baseline data, and educating participants. All 40 people who agreed to participate in this study made it through this session. However, as shown in Table 1, in the subsequent weeks that involved intervention sessions that were delivered over the telephone, we experienced a constant reduction in the number of people reached. For the final visit, which was again conducted in person, only 13 people were reachable.

**Table 1:** The number of participants who were reached via telephone.

Time point	Participants, n (%)
Week 2	25 (62)
Week 3	15 (37)
Week 4	13 (32)
Week 6	9 (22)

#### Data Collection

The pretest and posttest data were collected with the tablet computers that were provided to the EMS personnel during the in-person visits. The outcome assessment measures were embedded in the telehealth dashboard. This resulted in no missing or incomplete data, and little to no cleanup was required during the data analysis phase.

### Exploratory Measures

#### The FH

The FH survey results revealed a decreasing trend in the overall average scores for all three subdomains—the average scores for fear (mean 13.78, SD 6.3 vs mean 9.38, SD 1.19), avoidance (mean 8.19, SD 4.42 vs mean 6.08, SD 4.37), and interference (mean 10.97, SD 5 vs mean 7.92, SD 1.55). The sum of the scores in the pretest scale was 32.95. This decreased to 23.38 after the intervention. However, no significant decrease in posttest scale scores was identified (*P*=.90).

#### Self-efficacy

The one-tailed paired *t* test analysis revealed that the intervention resulted in significant improvements in participants’ self-efficacy in hypoglycemia self-management. Among the 13 participants who completed both the pre- and postsurvey, the average total PDSMS score significantly increased (mean 6, SD 8.59; *P*=.03). According to the results, the participants appeared to have significantly more confidence in the topic “No matter how hard I try, managing my diabetes doesn’t turn out the way I would like” and tended to exhibit an increase in scores for the “I am able to manage things related to my diabetes as well as most other people” topic. The scores for these topics increased by an average of 1.3 points (SD 1.11; *P*<.001) and 1 point (SD 1.73; *P*=.06), respectively.

#### Knowledge of Diabetes

The SKILLD survey results also showed improvements in participants’ knowledge of the complications of diabetes. The mean difference between the pre- and postsurvey scores was 0.5 (SD 0.65; *P*=.01). Although not significant, improvements in knowledge regarding symptoms of hypoglycemia (*P*=.33) and in normal hemoglobin A_1c_ levels (*P*=.27) were seen in the posttest scores.

## Discussion

### Study Overview

To our knowledge, this is the first study to conduct a community paramedicine and mobile integrated health care intervention [34] with a specific focus on hypoglycemia by using a telehealth platform. Working with a community partner enabled us to establish a smooth enrollment process for the intervention. However, the low retention rate was an important problem during intervention delivery and data collection. These should be explored in the context of this study to better understand and interpret the intervention outcomes.

### Factors That Lead to Low Retention

Residents of the area that was served by this project belong to the lowest quartile of health literacy scores [16]. Although we included 40 participants, relatively high dropout rates and low attendance rates were observed due to the participants’ low economic and educational statuses. As noted by our community health partner, most residents do not have a permanent phone number and use pay-as-you-go phones. Although participants were available for the baseline visit in week 1, they were not reachable for the final visit in week 8 for scheduling an in-person appointment. Tracking the participants via telephone was, by itself, challenging, and the lack of a permanent phone number made the process even more complicated. The results of this study show early promise, despite the challenging environment of the intervention. However, repeating this study in other socioeconomic settings or neighborhoods is needed to evaluate the intervention for its fullest potential. Additionally, other strategies for improving patient outreach and retention are needed to test this type of intervention.

### Study Personnel

This study helped us learn about several other aspects about the involvement of EMS personnel in community paramedicine programs. During our intervention, we experienced some challenges with completing the in-person visits. Due to safety concerns, EMS personnel carried out the visits at their convenience. Relying on EMS personnel hindered the collection of the data and the completion of this study. Additionally, because of the nature of this pilot study, we were unable to have exclusive staff join the EMS personnel during the in-person visits. Moreover, the EMS staff in this study changed several times during the intervention due to organizational reasons. Future studies should consider including exclusive staff to ensure protocol fidelity. Another solution is employing the temporarily injured employees of fire departments. Employees who are unable to actively return to fieldwork are often available in fire stations and are best suited for telehealth calls. Future research should consider using protocols for including injured EMS personnel who could actively participate as health coaches in studies.

### Importance of Trust

Another valuable insight we learned from this intervention was about the impact that EMS personnel’s attire had on the participants’ confidence and trust. We observed that wearing a professional uniform favorably influenced participants’ trust and confidence in the paramedics. Participants also expressed the most confidence when the same EMS personnel attended to their emergency medical needs. Future studies that try to evaluate community paramedicine or telehealth need to ensure that such factors of trust are considered in the intervention design.

We designed a telehealth dashboard that the EMS personnel in this study could use to coach and monitor participants with hypoglycemia over the phone. At the end of the 8-week intervention period, nonsignificant improvements were found across various knowledge domains and subdomains (fear: *P*=.74; avoidance: *P*=.60; interference: *P*=.88) of the FH. The intervention resulted in significant improvements in participants’ self-efficacy in hypoglycemia self-management and improved their knowledge of the complications of diabetes, which was measured by using the SKILLD scale. The lack of significant FH-related results might have been due to the very low completion rate (retention), as only 13 out of the 40 participants had posttest data. To interpret the results of this study in the right context, the challenges faced and the lessons learned during this study must be considered.

Although hypoglycemia can usually be safely and cost-effectively treated by paramedics, EMS protocols have been developed independently. This has led to variations in protocol content and formats, which can result in varying standards of care. However, the clinical practices of paramedics and emergency care protocols should be evidence-based and reflect common standards of care, formats, and content [35]. A standardized protocol can be easily accessed through a telehealth platform and can be used to provide guidance and education to patients. Therefore, by using a platform with evidence-based content and a standardized protocol, our study established an example of a telehealth-supported community service that can equally benefit people in need.

### Limitations

Although we wanted to conduct a randomized feasibility study, the establishment of an untreated control group was not accepted by our community partner due to ethical concerns. Therefore, we chose a pre-post study design. Future studies should consider using designs that involve either simple randomization (by individuals) trials or cluster randomization (by fire districts) trials to understand our intervention’s broader impacts. Although this pilot study was limited to a sample size of 40, future studies should also consider having larger sample sizes. Given the high attrition and the challenging socioeconomic settings in which this study was conducted, the findings of this study cannot be generalized. Similar studies should be conducted across areas with different socioeconomic populations. Finally, we offered health kits that were comprised of blood glucose test kits, blood pressure cuffs, and dextrose gels. However, it should be noted that these kits could have had a confounding effect on the outcomes of this study, as there is evidence suggesting that the mere presence of self-monitoring equipment can influence diabetes-related outcomes [29].

### Conclusions

Our study shows early promising results for a community-based hypoglycemia prevention intervention. However, our pilot study has several limitations. We comprehensively presented the challenges we faced and the lessons we learned throughout this study, and these should be considered when designing future studies.

## Abbreviations

DLNET: Diabetes Literacy and Numeracy Education Toolkit
EMS: emergency medical services
FH: fear of hypoglycemia
PDSMS: Perceived Diabetes Self-Management Scale
SKILLD: Spoken Knowledge in Low Literacy Diabetes

## References

[1] Kumar JG, Abhilash KPP, Saya RP, Tadipaneni N, Bose JM (2017). A retrospective study on epidemiology of hypoglycemia in emergency department. Indian J Endocrinol Metab.

[2] Moisan J, Breton MC, Villeneuve J, Grégoire JP (2013). Hypoglycemia-related emergency department visits and hypoglycemia-related hospitalizations among new users of antidiabetes treatments. Can J Diabetes.

[3] Thompson AE (2015). JAMA patient page. Hypoglycemia. JAMA.

[4] Malouf R, Brust JCM (1985). Hypoglycemia: causes, neurological manifestations, and outcome. Ann Neurol.

[5] Donnelly LA, Morris AD, Frier BM, Ellis JD, Donnan PT, Durrant R, Band MM, Reekie G, Leese GP, DARTS/MEMO Collaboration (2005). Frequency and predictors of hypoglycaemia in Type 1 and insulin-treated Type 2 diabetes: a population-based study. Diabet Med.

[6] Davis RE, Morrissey M, Peters JR, Wittrup-Jensen K, Kennedy-Martin T, Currie CJ (2005). Impact of hypoglycaemia on quality of life and productivity in type 1 and type 2 diabetes. Curr Med Res Opin.

[7] Desouza CV, Bolli GB, Fonseca V (2010). Hypoglycemia, diabetes, and cardiovascular events. Diabetes Care.

[8] Laing SP, Swerdlow AJ, Slater SD, Botha JL, Burden AC, Waugh NR, Smith AWM, Hill RD, Bingley PJ, Patterson CC, Qiao Z, Keen H (1999). The British Diabetic Association Cohort Study, II: cause-specific mortality in patients with insulin-treated diabetes mellitus. Diabet Med.

[9] Wild D, von Maltzahn R, Brohan E, Christensen T, Clauson P, Gonder-Frederick L (2007). A critical review of the literature on fear of hypoglycemia in diabetes: Implications for diabetes management and patient education. Patient Educ Couns.

[10] Fidler C, Christensen TE, Gillard S (2011). Hypoglycemia: an overview of fear of hypoglycemia, quality-of-life, and impact on costs. J Med Econ.

[11] Shepard JA, Vajda K, Nyer M, Clarke W, Gonder-Frederick L (2014). Understanding the construct of fear of hypoglycemia in pediatric type 1 diabetes. J Pediatr Psychol.

[12] Shaefer C, Hinnen D, Sadler C (2016). Hypoglycemia and diabetes: increased need for awareness. Curr Med Res Opin.

[13] Griffey RT, Kennedy SK, McGowan LD, Goodman M, Kaphingst KA (2014). Is low health literacy associated with increased emergency department utilization and recidivism?. Acad Emerg Med.

[14] Sarkar U, Karter AJ, Liu JY, Moffet HH, Adler NE, Schillinger D (2010). Hypoglycemia is more common among type 2 diabetes patients with limited health literacy: the Diabetes Study of Northern California (DISTANCE). J Gen Intern Med.

[15] Vernon J, Trujillo A, Rosenbaum S, DeBuono B (2007). Low health literacy: Implications for national health policy. Semantic Scholar.

[16] Health literacy data map. The University of North Carolina at Chapel Hill.

[17] Prezio EA, Cheng D, Balasubramanian BA, Shuval K, Kendzor DE, Culica D (2013). Community diabetes education (CoDE) for uninsured Mexican Americans: a randomized controlled trial of a culturally tailored diabetes education and management program led by a community health worker. Diabetes Res Clin Pract.

[18] Culica D, Walton J, Prezio E (2007). CoDE: Community diabetes education for uninsured Mexican Americans. Proc (Bayl Univ Med Cent).

[19] Davidson MB, Ansari A, Karlan VJ (2007). Effect of a nurse-directed diabetes disease management program on urgent care/emergency room visits and hospitalizations in a minority population. Diabetes Care.

[20] Bennett KJ, Yuen MW, Merrell MA (2018). Community paramedicine applied in a rural community. J Rural Health.

[21] Choi BY, Blumberg C, Williams K (2016). Mobile integrated health care and community paramedicine: An emerging emergency medical services concept. Ann Emerg Med.

[22] Zavadsky M, Hagen T, Hinchey P, McGinnis K, Bourn S, Myers B (2015). Mobile integrated health care and community paramedicine (MIH-CP). National Association of Emergency Medical Technicians.

[23] Kizer KW, Shore K, Moulin A (2013). Community paramedicine: A promising model for integrating emergency and primary care. UC Davis Institute for Population Health Improvement.

[24] Misner D (2005). Community paramedicine: part of an integrated healthcare system. Emerg Med Serv.

[25] Moffet HH, Warton EM, Siegel L, Sporer K, Lipska KJ, Karter AJ (2017). Hypoglycemia patients and transport by EMS in Alameda County, 2013-15. Prehosp Emerg Care.

[26] Geller AI, Shehab N, Lovegrove MC, Kegler SR, Weidenbach KN, Ryan GJ, Budnitz DS (2014). National estimates of insulin-related hypoglycemia and errors leading to emergency department visits and hospitalizations. JAMA Intern Med.

[27] Liatis S, Mylona M, Kalopita S, Papazafiropoulou A, Karamagkiolis S, Melidonis A, Xilomenos A, Ioannidis I, Kaltsas G, Lanaras L, Papas S, Basagiannis C, Kokkinos A (2015). Hypoglycaemia requiring medical assistance in patients with diabetes: a prospective multicentre survey in tertiary hospitals. Diabetes Metab.

[28] Gabbay RA, Kahn PA, Wagner NE (2018). Underutilization of glucagon in the prehospital setting. Ann Intern Med.

[29] Sarkar U, Fisher L, Schillinger D (2006). Is self-efficacy associated with diabetes self-management across race/ethnicity and health literacy?. Diabetes Care.

[30] Wolff K, Cavanaugh K, Malone R, Hawk V, Gregory BP, Davis D, Wallston K, Rothman RL (2009). The Diabetes Literacy and Numeracy Education Toolkit (DLNET): materials to facilitate diabetes education and management in patients with low literacy and numeracy skills. Diabetes Educ.

[31] Rothman RL, Malone R, Bryant B, Wolfe C, Padgett P, DeWalt DA, Weinberger M, Pignone M (2005). The spoken knowledge in low literacy in diabetes scale: a diabetes knowledge scale for vulnerable patients. Diabetes Educ.

[32] Ortiz MTA, Caballero FF, de Adana MSR, Rondán RM, Carreira M, Domínguez-López M, Machado A, Gonzalo-Marín M, Tapia MJ, Valdés S, González-Romero S, Soriguer FC (2011). Development of a new fear of hypoglycemia scale: FH-15. Psychol Assess.

[33] Wallston KA, Rothman RL, Cherrington A (2007). Psychometric properties of the Perceived Diabetes Self-Management Scale (PDSMS). J Behav Med.

[34] Gregg A, Tutek J, Leatherwood MD, Crawford W, Friend R, Crowther M, McKinney R (2019). Systematic review of community paramedicine and EMS mobile integrated health care interventions in the United States. Popul Health Manag.

[35] Anantharaman G (2004). Standards and standardization in paramedic protocols. Australasian Journal of Paramedicine.

